# Can Friendship Quality Buffer the Impact of Parental Phubbing on Adolescents’ Gratitude? The Longitudinal Mediating Role of Basic Psychological Needs’ Satisfaction

**DOI:** 10.3390/bs14111083

**Published:** 2024-11-12

**Authors:** Bowen Lu, Xinyuan Shen, Xiaosong Gai, Xiaochun Xie

**Affiliations:** 1 School of Psychology, Northeast Normal University, Changchun 130024, China; lubw605@nenu.edu.cn (B.L.); shenxy992@nenu.edu.cn (X.S.); gaixs669@nenu.edu.cn (X.G.); 2 Research Center of Mental Health Education in Northeast Normal University, Key Research Institute of Humanities and Social Science in Universities in Jilin Province, Changchun 130024, China

**Keywords:** adolescents, parental phubbing, gratitude, basic psychological needs’ satisfaction, friendship quality

## Abstract

This study aims to explore the longitudinal relationship between parental phubbing and adolescents’ gratitude, as well as the mediating role of basic psychological needs’ satisfaction and the moderating role of friendship quality. We conducted this longitudinal study in two waves with a 7-month, and surveyed 643 Chinese adolescents and constructed a moderated mediation model. The results indicated the following: First, there is a significant negative correlation between parental phubbing and adolescents’ gratitude. Second, adolescents’ basic psychological needs’ satisfaction mediates the relationship between parental phubbing and gratitude. Finally, friendship quality moderates the negative relationship between parental phubbing and adolescents’ basic psychological needs’ satisfaction and the mediating effect of basic psychological needs’ satisfaction. Specifically, compared to adolescents with lower friendship quality, the negative effect of parental phubbing on basic psychological needs’ satisfaction is stronger among adolescents with higher friendship quality, and the negative indirect effect of parental phubbing on gratitude through basic psychological needs’ satisfaction is also stronger in these adolescents. The findings suggest that parental phubbing is a significant risk factor for decreased adolescents’ gratitude, with high friendship quality adolescents being more susceptible to the impact of parental phubbing. The negative impact of parental phubbing outweighs the influence of friendship quality. This study provides insights into interventions promoting adolescents’ positive development.

## 1. Introduction

Gratitude is a positive emotional experience that arises when an individual recognizes the benefits received from others and responds with appreciative emotions [1]. As one of the 24 positive psychological traits in adolescents [2], gratitude is a crucial positive emotion in the development of adolescents [3]. Adolescents with higher gratitude often exhibit more positive coping attitudes and strategies when facing risks and challenges [4]. Research has shown that gratitude benefits adolescents’ psychological health and positive development and can enhance their well-being [5,6,7]. Family factors are significant in influencing adolescents’ gratitude. Positive parenting can increase adolescents’ gratitude [8]. The parent–child relationship is crucial in cultivating adolescents’ gratitude [9,10]. Studies indicate that a positive parent–child relationship significantly and positively predicts adolescents’ gratitude [11,12]. In contrast, a negative parent–child relationship significantly and negatively predicts adolescents’ gratitude [13].

In the context of the rapid advancement of internet technology and the widespread adoption of smartphones, there is growing concern about the issue of phubbing caused by phone dependency. A study examining data from 2019 to 2022 on a sample of 10,048 U.S. adolescents aged 12 to 13 found that 72.9% of parents used screen-based devices, such as mobile phones, in the presence of their children [14]. The use of phones can lead individuals to neglect those around them, particularly in parent–child interactions. Frequent phone use by parents inevitably reduces communication with their children [15], thereby ignoring their emotional needs. This indicates that phone use has become intertwined with parenting processes. Research shows that parental phubbing negatively impacts the quality of parent–child relationships [16,17], leading to the internalization of psychological issues such as anxiety and depression in children and the externalization of behavior problems such as aggression and internet addiction [18,19,20]. However, no studies have directly addressed parental phubbing’s impact on adolescents’ gratitude.

In summary, this study employs a longitudinal research design, focusing on middle- and high-school students, to elucidate the mechanisms through which parental phubbing affects gratitude among Chinese adolescents. This study deepens the understanding of the relationship between parental phubbing and adolescents’ gratitude by identifying the moderating effects of friendship quality on this relationship. Specifically, we explore how parental phubbing decreases adolescents’ gratitude through basic psychological needs’ satisfaction. Furthermore, we elucidate when the effect of parental phubbing on adolescents’ gratitude is pronounced or diminished, depending on the level of friendship quality.

### 1.1. Parental Phubbing and Gratitude

Phubbing refers to the behavior of interrupting interpersonal communication due to smartphone use, resulting in the interaction partner feeling neglected [21,22]. Parental phubbing explicitly describes a situation where parents, through their use of phones, neglect communication with their children during family interactions, causing the children to feel overlooked [23]. Parental phubbing can be seen as a form of social exclusion [24], conveying to children the message that the importance of the phone outweighs their significance [25]. According to the Parental Acceptance–Rejection Theory, when children’s emotional needs are unmet by their parents, they may experience a sense of rejection, leading to various psychological adjustment issues [26,27]. Research indicates that parental phubbing can trigger the internalization of psychological problems such as anxiety and depression in children and the externalization of behavior problems such as aggression and internet addiction [18,19,20]. Additionally, parental phubbing deteriorates the parent–child relationship quality [16] and harms parent–child attachment [28]. The time parents invest in phone use reduces the time available for parent–child interactions [15,29], thereby negatively impacting the parent–child relationship [17,30].

The parent–child relationship plays a crucial role in developing adolescents’ gratitude [9]. A positive parent–child relationship significantly and positively predicts adolescents’ gratitude [11,12], while a negative parent–child relationship significantly and negatively predicts adolescents’ gratitude [13]. Research has shown that parental phubbing significantly and positively predicts adolescents’ perceptions of parental rejection [31]. The higher the perceived parental rejection, the lower the adolescents’ gratitude [32]. The Parental Acceptance–Rejection Theory suggests that, after perceiving parental rejection, adolescents find it hard to express love to others [27]. Therefore, based on the Parental Acceptance–Rejection Theory, this study hypothesizes that parental phubbing has a negative impact on adolescents’ gratitude. This is because adolescents experiencing parental phubbing may feel rejected, excluded, and neglected, which negatively affects the parent–child relationship and, in turn, reduces adolescents’ gratitude. Consequently, this study proposes Hypothesis 1 (H1): Parental phubbing would have a negative correlation with adolescents’ gratitude.

### 1.2. The Mediating Role of Basic Psychological Needs’ Satisfaction

The self-determination theory posits that humans have three innate basic psychological needs: autonomy, competence, and relatedness. This theory suggests that fulfilling these basic psychological needs promotes positive development, while unmet needs undermine the intrinsic motivation required for healthy development [33]. The self-determination theory posits that basic psychological needs can be understood both broadly and in the context of specific interpersonal interactions, where individuals derive satisfaction of these needs through their relationships with others. Individuals tend to seek relationships, and when these relationships provide opportunities for basic psychological needs’ satisfaction, they can experience a sense of well-being within those relationships. One study revealed that basic psychological needs’ satisfaction experienced in specific relationships, such as parent–child relationships, romantic relationships, and peer relationships, can predict a secure attachment to relative partners in those relationships [34]. Additionally, a study has explored that basic psychological needs’ satisfaction increased perceived well-being within friendships [35]. The current study aimed to investigate how parenting behavior impacts adolescents’ basic psychological needs’ satisfaction.

The family environment plays a crucial role in adolescent development [36]. A need-supportive environment can facilitate positive development by meeting these basic psychological needs [37]. A positive family environment can enhance adolescents’ basic psychological needs’ satisfaction. As a form of need-supportive parenting, parental support of autonomy significantly positively predicts adolescents’ basic psychological needs’ satisfaction [38]. Conversely, a negative family environment and maladaptive parenting practices can undermine adolescents’ basic psychological needs’ satisfaction. Parental rejection significantly positively predicts the thwarting of adolescents’ basic psychological needs [39]. Parental phubbing, being a form of rejection and neglect, leads adolescents to feel excluded by their parents, thereby reducing their sense of relatedness satisfaction [31]. Therefore, this study hypothesizes that parental phubbing negatively impacts adolescents’ basic psychological needs’ satisfaction, which in turn affects their gratitude.

Gratitude is an essential positive emotion in the developmental process of adolescents [3], and basic psychological needs’ satisfaction plays a crucial role in enhancing positive emotions [40]. Parental support for autonomy significantly positively predicts children’s gratitude, as this supportive parenting approach satisfies children’s basic psychological needs. Hence, gratitude can increase basic psychological needs’ satisfaction [41]. Previous research has identified a significant positive correlation between gratitude and autonomy [42], with experiences of gratitude fulfilling the basic psychological need for autonomy [43]. Furthermore, greater basic psychological needs’ satisfaction can further promote greater gratitude [44]. Thus, this study hypothesizes that basic psychological needs’ satisfaction positively promotes gratitude. In summary, parental phubbing may affect adolescents’ gratitude by diminishing their basic psychological needs’ satisfaction. Therefore, this study proposes Hypothesis 2 (H2): Adolescents’ basic psychological needs’ satisfaction would mediate the relationship between parental phubbing and gratitude.

### 1.3. The Moderating Role of Friendship Quality

Friendship is a peer relationship based on mutual attraction and the principle of equal social exchange [45]. Friendship quality, a key indicator for evaluating the strength of a friendship, reflects the level of support, companionship, and conflict within the relationship [46]. Studies have shown that high-quality friendships can significantly enhance adolescents’ sense of self-worth, interpersonal skills [47], and self-esteem [48]. During adolescence, the influence of peers increasingly surpasses that of parents, becoming the primary source for fulfilling individual needs [49,50]. According to the friendship protection hypothesis, friends’ support may help buffer negative experiences’ effects and mitigate their impact [51]. Therefore, adolescents experiencing parental phubbing may turn to their friends for comfort and support. High friendship quality can satisfy individuals’ basic psychological needs [52,53,54]. Research has demonstrated that friendship quality significantly and positively predicts adolescents’ basic psychological needs’ satisfaction [55]. Both parenting styles and friendship quality are critical predictors of adolescents’ basic psychological needs’ satisfaction [56]. We suppose that friendship quality can moderate the relationship between parental phubbing and adolescents’ basic psychological needs’ satisfaction.

**Figure 1:**
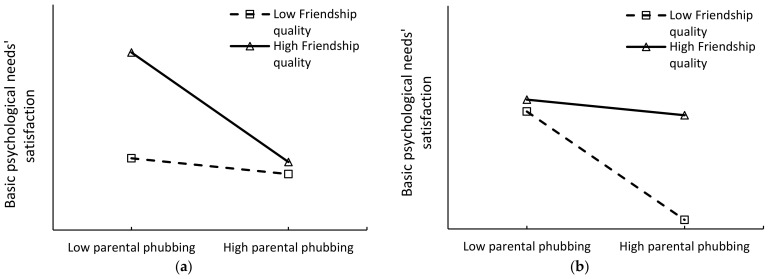
(**a**) Schematic diagram of H3a. (**b**) Schematic diagram of H3b.

The moderating role of friendship quality may exhibit two distinct effects: reinforcement and compensation. Specifically, the reinforcement effect suggests that higher friendship quality exacerbates the negative impact of parental phubbing on adolescents’ basic psychological needs’ satisfaction. Research has indicated that high friendship quality can significantly amplify the influence of negative parent–child relationships on adolescents’ depressive moods [57]. In contrast, the compensation effect implies that friendship quality can mitigate or buffer the negative impact of parental phubbing on adolescents’ basic psychological needs’ satisfaction. Studies have shown that high friendship quality can buffer the effects of negative parent–child relationships on adolescents’ problem behaviors [58]. Accordingly, this study proposes the following two competing hypotheses to examine the moderating role of friendship quality: Hypothesis 3a (H3a): Friendship quality would positively moderate the negative relationship between parental phubbing and adolescents’ basic psychological needs’ satisfaction, meaning that higher friendship quality would strengthen this negative relationship (see Figure 1a). Hypothesis 3b (H3b): Friendship quality would negatively moderate the negative relationship between parental phubbing and adolescents’ basic psychological needs’ satisfaction, meaning that higher friendship quality would weaken this negative relationship (see Figure 1b).

**Figure 2:**
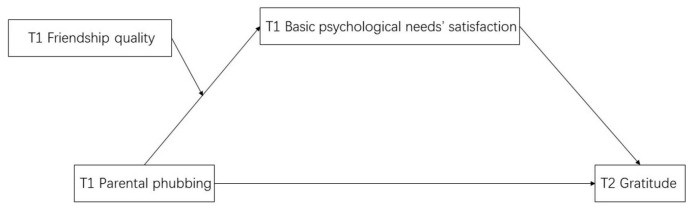
Hypothetical model of the relationships among parental phubbing, basic psychological needs’ satisfaction, friendship quality, and gratitude.

Integrating H2 and H3, this study constructs a moderated mediation model (see Figure 2) and proposes two complementary hypotheses based on this model. Hypothesis 4a (H4a): Friendship quality would positively moderate the negative indirect effect of parental phubbing on adolescents’ gratitude through adolescents’ basic psychological needs’ satisfaction, meaning that higher friendship quality would strengthen this negative indirect effect. Hypothesis 4b (H4b): Friendship quality would negatively moderate the negative indirect effect of parental phubbing on adolescents’ gratitude through adolescents’ basic psychological needs’ satisfaction, meaning that higher friendship quality would weaken this negative indirect effect.

## 2. Methods

### 2.1. Procedures

This analysis draws on the first (T1) and second (T2) annual data points of the longitudinal cohort study sample (from a middle school and a high school in the northeast of China). The data for this study were collected at two different time points: April 2021 and November 2021. The T1 longitudinal sample (n = 810) consists of adolescents in grades 7–8. Parental phubbing, basic psychological needs’ satisfaction, friendship quality, and gratitude were drawn from T1 survey data. T2 was carried out seven months later and consisted of T1 participants still enrolled in school. Gratitude was drawn from T2 survey data. The questionnaire included an item where participants self-reported their level of attentiveness: “To what extent did you carefully answer the questions?” (scored from 1 to 10). Six hundred forty-three valid questionnaires were obtained after missing data and careless responses were eliminated. The retention rate from T1 to T2 was 79.38%. Participants provided informed consent before completing the survey. The class teachers supervised the survey process. The instructions for the questionnaire assured participants that their information would be kept confidential. The questionnaire was administered in Chinese. The average completion time was 15 min. This study was approved by the Research Ethics Committee of the author’s affiliated institution (protocol code 2024012).

### 2.2. Participants

The valid sample included 643 participants, of which 301 were male (46.81%), and 329 were female (51.17%), with gender data missing for 13 participants (2.02%). There were 343 middle-school students (53.34%), including 205 in the second year and 138 in the third year. There were 299 high-school students (46.50%), including 141 in the second year and 158 in the third year. Grade data were missing for 1 participant (0.16%). Participants’ ages ranged from 12 to 18 years, with a mean age of 14.91 ± 1.56 years.

### 2.3. Measures

#### 2.3.1. Parental Phubbing

The Chinese version Parental Phubbing Scale [21,23] was employed to measure adolescents’ perceptions of parental phubbing. The scale consists of nine items (e.g., “When our family spends leisure time together, my parents look at their phones”). The items are rated on a 5-point Likert-type response format, ranging from 1 = Never to 5 = Always, with higher scores meaning more parental phubbing. The scale had a Cronbach’s alpha of 0.83 in this sample.

#### 2.3.2. Basic Psychological Needs’ Satisfaction

The Basic Psychological Needs’ Satisfaction in Relationships Scale [34] was used to measure the participants’ perceived basic psychological needs’ satisfaction. This scale was translated to the Chinese version and tested for good reliability and validity in Chinese adolescents in a previous study [59]. This study replaced the original items with “When I am with my parents” with the phrase “When I am with a person”. The scale comprises nine items, encompassing three dimensions: autonomy (e.g., “When I am with my parents, I feel free to be who I am”), competence (e.g., “When I am with my parents, I feel like a competent person”), and relatedness (e.g., “When I am with my parents, I feel loved and cared for”), with each dimension represented by three items. The items are rated on a 7-point Likert-type response format, ranging from 1 = Not at all true to 7 = Very true, with higher scores meaning more basic psychological needs’ satisfaction. The scale had a Cronbach’s alpha of 0.86 in this sample.

#### 2.3.3. Gratitude

The Chinese version Gratitude Questionnaire (GQ-6) [1,60] was used to measure adolescents’ gratitude. The scale consists of six items (e.g., “I have so much in life to be thankful for”). The items are rated on a 6-point Likert-type response format, ranging from 1 = Strongly disagree to 6 = Strongly agree, with higher scores meaning more gratitude. The scale had a Cronbach’s alpha of 0.81 in this sample.

#### 2.3.4. Friendship Quality

The Friendship Quality Questionnaire (FQQ) was a short Chinese language-adapted version of the 40-item Friendship Quality Scale [46,61], which measured friendship quality with a best friend in this study to avoid ambiguity when the participants assessed their friendship quality. The FQQ comprises eighteen items that cover six dimensions: Validation and Caring (e.g., “My friend tells me I am good at things”), Help and Guidance (e.g., “We often help each other with homework”), Companionship and Recreation (e.g., “We spend time together whenever we get the chance”), Intimate Exchange (e.g., “We talk about things that make us sad”), Conflict Resolution (e.g., “My friend often gives me advice on how to solve problems”), and Conflict and Betrayal (e.g., “We often argue”). Each dimension includes three items. The items are rated on a 5-point Likert-type response format, ranging from 1 = Not at all true to 5 = Completely true, with higher scores meaning more friendship quality. The scale had a Cronbach’s alpha of 0.87 in this sample.

### 2.4. Data Analysis

We analyzed data using IBM SPSS software version 26.0. The missing data in the analytical sample were 23%, which were accounted for using the Expectation–Maximization (EM) algorithm. The analyses were carried out in three steps. First, common method bias was tested using Harman’s single-factor test. Second, descriptive statistics and correlation analyses were conducted for all variables. Finally, to test the mediation and moderated mediation hypotheses, we used Models 4 and 7, respectively, in the PROCESS macro developed by Hayes [62]. The indirect effects with bias-corrected bootstrapping (n = 5000) and confidence intervals (CIs) for indices were employed. Parameter estimates were significant if the bootstrapped CIs at 95% did not include zero. An index of moderated mediation was calculated with confidence intervals. All variables were standardized before conducting the mediation and moderation analyses.

## 3. Results

### 3.1. Test for Common Method Bias

This study utilized self-report questionnaires to collect data. To avoid the issue of common method bias, we informed the participants that their survey responses would re-main anonymous. Additionally, different scales employed varied scoring methods (e.g., five-point, six-point, and seven-point scales), and some items were reverse-scored. Harman’s single-factor test was employed to assess common method bias [63]. The results indicated that there were 11 factors with eigenvalues greater than 1, and the first factor accounted for 21% of the total variance, below the critical threshold of 40%. Therefore, this study does not exhibit significant common method bias.

### 3.2. Descriptive Statistics

T1 parental phubbing was significantly negatively correlated with T1 basic psychological needs’ satisfaction (r = −0.45, *p* < 0.01). T1 parental phubbing was significantly negatively correlated with T2 gratitude (r = −0.21, *p* < 0.01). T1 basic psychological needs’ satisfaction was significantly positively correlated with T2 gratitude (r = 0.35, *p* < 0.01). These findings confirm Hypothesis 1. For a complete description of the sample and the bivariate correlations, see Table 1.

### 3.3. Mediation Effect of Basic Psychological Needs’ Satisfaction

T1 parental phubbing was used as the independent variable, T2 gratitude as the dependent variable, and T1 basic psychological needs’ satisfaction as the mediator, with T1 gratitude included as a control variable in the mediation models (see Table 2). The effect of T1 parental phubbing on T1 basic psychological needs’ satisfaction was negative and significant (β = −0.37, *p* < 0.001). The effect of T1 basic psychological needs’ satisfaction on T2 gratitude was positive and significant (β = 0.13, *p* < 0.01). The direct effect of T1 parental phubbing on T2 gratitude was insignificant (β = −0.05, *p* = 0.16). The standardized indirect effect of T1 parental phubbing on T2 gratitude through T1 basic psychological needs’ satisfaction was B = −0.05, SE = 0.02, 95% *CI* = [−0.09, −0.01]. The mediation effect accounted for 50% of the total effect. T1 basic psychological needs’ satisfaction mediates the link between T1 parental phubbing and T2 gratitude, confirming Hypothesis 2.

### 3.4. Moderated Mediation Effects of Gratitude

T1 parental phubbing was used as the independent variable, T2 gratitude as the dependent variable, T1 basic psychological needs’ satisfaction as the mediator, and T1 friendship quality as the moderator, with T1 gratitude included as a control variable in the moderated mediation models (see Table 3). The results revealed that T1 friendship quality moderated the associations between T1 parental phubbing and T1 basic psychological needs’ satisfaction β = −0.07, SE = 0.03, 95% *CI* = [−0.13, −0.02].

**Figure 3:**
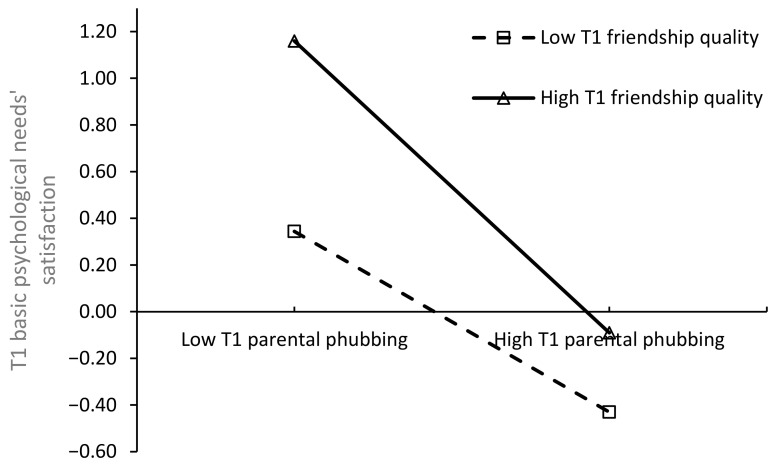
The moderating effect of T1 friendship quality on the relationship between T1 parental phubbing and T1 basic psychological needs’ satisfaction.

Simple slope tests were used to interpret the relationship (see Figure 3). The results revealed that at high (*M* + 1*SD*) [β simple = −0.43, *t* = −10.22, *p* < 0.001] and low (*M* − 1*SD*) [β simple = −0.28, *t*= −6.17, *p* < 0.001] T1 friendship quality, the effect of T1 parental phubbing on T1 basic psychological needs’ satisfaction was negative and significant (see Table 4). This indicates that as T1 friendship quality increases, the negative predictive effect of T1 parental phubbing on T1 basic psychological needs’ satisfaction intensifies, confirming Hypothesis 3a.

The bias-corrected percentile bootstrap method and resulting index of moderated mediation further confirmed the significant moderated mediation effect, β = −0.010, SE = 0.005, 95% *CI* = [−0.022, −0.001], where T1 friendship quality moderated the first stage of the mediating relationship from T1 parental phubbing to T1 basic psychological needs’ satisfaction to T2 gratitude. The indirect effect of T1 parental phubbing on T2 gratitude through T1 basic psychological needs’ satisfaction was statistically significant for adolescents with high (*M* + 1*SD*) β= −0.055, SE = 0.022, 95% *CI* = [−0.103, −0.015] and low (*M* − 1*SD*) β= −0.036, SE = 0.016, 95% *CI* = [−0.071, −0.009] T1 friendship quality (see Table 5). Specifically, the mediation effect of T1 basic psychological needs’ satisfaction in the relationship between T1 parental phubbing and T2 gratitude is significantly greater for students with higher T1 friendship quality than those with lower T1 friendship quality. Friendship quality positively moderates the negative indirect effect of parental phubbing on adolescent gratitude, meaning that higher friendship quality strengthens this negative indirect effect. These findings confirm Hypothesis 4a.

## 4. Discussion

This study examined the mechanisms through which parental phubbing affects adolescents’ gratitude, specifically investigating the mediating role of basic needs’ satisfaction and the moderating role of friendship quality. The findings revealed a significant negative correlation between parental phubbing and adolescents’ gratitude. Basic psychological needs’ satisfaction was found to fully mediate the relationship between parental phubbing and adolescents’ gratitude, while friendship quality moderated the first part of this mediation pathway. The innovation of this study lies in expanding the existing research on the relationship between parental phubbing and adolescents’ gratitude, and in revealing how friendship quality enhances the negative association between parental phubbing and adolescents’ basic psychological needs’ satisfaction and gratitude.

### 4.1. The Relationship Between Parental Phubbing and Adolescents’ Gratitude

The results of this study indicate a significant negative correlation between parental phubbing and adolescents’ gratitude. Parental phubbing does not directly cause changes in adolescents’ gratitude but may indirectly influence their gratitude attitudes through other factors. Research has shown that parent–child relationships are crucial in developing adolescents’ gratitude [9]. Positive parent–child relationships significantly predict higher adolescents’ gratitude [11,12], while negative parent–child relationships significantly predict lower gratitude [13]. However, parental phubbing may undermine the parent–child relationship. Specifically, parental phubbing can reduce the frequency of parent–child communication [15], and partner phubbing among parents can affect marital intimacy [64], which, in turn, affects the overall quality of family relationships. Furthermore, according to the Parental Acceptance–Rejection Theory [26], parental phubbing may lead children to feel neglected, rejected, and excluded [31], thereby damaging the quality of the parent–child relationship [16], causing parent–child conflicts [65], and negatively impacting adolescents’ gratitude. This emotional estrangement can lead to the internalization of issues such as depression [18] and even result in the externalization of problems such as aggressive behavior [19]. Additionally, children often imitate parental phubbing behaviors, increasing their screen time, which may lead to internet addiction [20].

Overall, parental phubbing is a significant risk factor for maladaptive behaviors and issues in adolescents, negatively impacting their gratitude. The family environment and parent–child relationships are crucial for adolescents’ emotional development [36]. Therefore, within the family context, parents should reduce the time spent using their phones and increase face-to-face interactions with their children to foster better parent–child relationships, enhance children’s sense of gratitude, and promote their positive and healthy development.

### 4.2. The Role of Basic Psychological Needs’ Satisfaction

The analysis revealed a complete mediation effect, with parental phubbing not having a significant direct impact on gratitude but exerting a significant indirect effect through basic psychological needs’ satisfaction. On the one hand, parental phubbing reduces adolescents’ basic psychological needs’ satisfaction. This finding aligns with previous research [59,66], likely because parental phubbing leads adolescents to perceive parental rejection, resulting in decreased basic psychological needs’ satisfaction [31]. On the other hand, this study also found that adolescents’ basic psychological needs’ satisfaction can longitudinally predict an increase in gratitude seven months later. This is consistent with prior research showing that higher basic psychological needs’ satisfaction promotes increased gratitude [44]. Research indicates that the satisfaction of autonomy, relatedness, and competence needs can lead to higher levels of positive emotions [40]. As a positive emotion [3], gratitude can be further enhanced by satisfying basic psychological needs. Thus, parental phubbing has enduring adverse effects, as it lowers basic psychological needs’ satisfaction, which predicts lower future gratitude in adolescents. Parental phubbing is undoubtedly a significant predictor of internalizing problems in adolescents, negatively impacting their emotional well-being and mental health. It represents a form of parenting behavior that thwarts adolescents’ psychological needs. Therefore, parents should adopt more autonomy-supportive parenting approaches to foster adolescents’ basic psychological needs’ satisfaction [38], thereby enhancing their gratitude.

### 4.3. The Role of Friendship Quality

This study found that friendship quality significantly moderates the relationship between parental phubbing and basic psychological needs’ satisfaction. Specifically, higher friendship quality strengthens the negative predictive effect of parental phubbing on adolescents’ basic psychological needs’ satisfaction within the parent–child relationship. Furthermore, friendship quality also moderates the negative indirect effect of parental phubbing on adolescents’ gratitude. That is, the higher the friendship quality, the greater the negative impact of parental phubbing on adolescents’ attitudes of gratitude. Friendship quality amplifies the adverse effects of parental phubbing on basic psychological needs’ satisfaction and gratitude in adolescents. This finding contrasts with previous research suggesting that high-quality friendships can compensate for the internalizing problems caused by poor parent–child relationships [67]. Peer relationships are characterized by parallel and equal standings, unlike the vertical nature of relationships between individuals and their parents [68]. This difference reflects varying power distances [69]. Therefore, peer relationships and parent–child relationships possess distinct attributes and cannot compensate for or replace each other.

This study found that friendship quality does not buffer the impact of parental phubbing on adolescents’ gratitude. On the contrary, adolescents with higher friendship quality are more susceptible to the effects of parental phubbing. Previous research has similarly indicated that adolescents with better peer relationships are more likely to experience negative feelings when subjected to harmful parenting practices [70,71,72]. The habituation model can be used to explain individuals’ perceptions of harmful stimuli in their family environment. Specifically, when individuals are chronically exposed to harmful stimuli in a hostile family environment, they become desensitized to emotional distress and emotionally numb to harmful interactions [73]. Emotional distress, often manifested as feelings of rejection or exclusion, triggers a cognitive deconstruction defensive state characterized by emotional numbness [74]. On the one hand, friendship quality, as an indicator of interpersonal relationship quality, is negatively correlated with negative parent–child relationships [75]. Adolescents with lower friendship quality are typically in poorer interpersonal and family environments, with a lower basic psychological needs’ satisfaction. They are more accustomed to rejection, making the negative impact of parental phubbing on basic psychological needs’ satisfaction and gratitude less pronounced. On the other hand, high friendship quality is associated with positive parent–child relationships and is closely linked to adolescent mental health [76,77]. Compared to adolescents with lower friendship quality, those with higher friendship quality exhibit higher basic psychological needs’ satisfaction and gratitude in the absence of parental phubbing. This indicates they are in a favorable interpersonal and family environment, fostering healthier psychosocial development. They are more sensitive to increased parental phubbing and feel more discomfort. Thus, negative parent–child interactions in the family are more harmful to adolescents with healthier psychological development. Conversely, adolescents in adverse family environments, even if experiencing frequent parental phubbing, show lower sensitivity due to habituation.

This suggests that reducing parental phubbing plays a crucial role in promoting the adolescents’ basic psychological needs’ satisfaction and enhancing their gratitude. The adverse effects of parental phubbing outweigh the influence of friendship quality. Therefore, intervention strategies should focus on decreasing parental phubbing to create a positive family environment and improve parent–child relationships.

### 4.4. Implications

Our current work illustrates how parental phubbing decreased adolescents’ gratitude through basic psychological needs’ satisfaction and the moderating role of friendship quality; the results have both theoretical and practical implications. From the theoretical perspective, first, given that a number of studies on the adverse effect of parental phubbing are focused on adolescents’ mental health (e.g., depression, anxiety, addiction, low self-esteem, etc.), our work expanded previous studies to illustrate that parental phubbing can undermine adolescents’ positive psychological trait (gratitude). Cultivating gratitude is an important socialization goal for children and adolescents [78]. Therefore, our findings may reveal that parental phubbing impedes adolescent socialization. Second, this study revealed a reversed-buffering effect of friendship quality. This indicates that peer relationships do not always effectively buffer the negative impact of parental phubbing on adolescents’ gratitude. Theoretically, this provides a boundary condition on the mechanism of the relation between parental phubbing and adolescent gratitude.

On practical implications, first, this study confirms that parental phubbing has a negative impact on adolescents’ social development. Given the crucial role of the family environment and parent–child relationships in adolescents’ emotional development [36], this research calls for parents to adopt positive parenting practices, such as autonomy support [38], during the parenting process. It is recommended that parents reduce their smartphone usage in front of their children and increase their attention towards them. Second, this study finds that although the direct impact of peer relationships is relatively limited, friendship quality plays a facilitating role in basic psychological needs’ satisfaction, indicating that adolescents’ friendships still hold significant importance. Furthermore, for adolescents with high friendship quality, the negative effects of parental phubbing are more pronounced in adverse parenting environments. Therefore, maintaining a good parent–child relationship should be prioritized for adolescents with high friendship quality. Lastly, schools can enhance parenting quality through educational outreach to parents, such as parent–teacher meetings and promoting collaboration between home and school.

### 4.5. Limitations and Future Research

This study collected data through self-reported questionnaires from participants, which may introduce certain common method biases and social desirability biases. Future research could consider using parental perceptions of adolescents’ gratitude as an indicator to enhance measurement accuracy. Additionally, this study did not differentiate parental phubbing by gender. Future research could investigate the effects and mechanisms of fathers’ and mothers’ phubbing behavior on adolescents’ gratitude separately. Moreover, the longitudinal tracking period in this study was only seven months, which is relatively short. Future studies could extend the tracking duration and include more measurement time points to increase the reliability of the results. Finally, the findings of this study indicated that friendship quality did not buffer the impact of parental phubbing on adolescents’ gratitude, possibly due to the limitations of the sample selection. The sample was restricted to the northeastern region of China, which may affect the generalizability and applicability of the results. Future research should consider expanding the sample to a broader geographical area and include adolescents from different educational stages to obtain more representative findings.

## 5. Conclusions

This study explored the relationship between parental phubbing and adolescents’ gratitude, the mediating role of basic psychological needs’ satisfaction and the moderating role of friendship quality. The findings revealed several key points: First, a significant negative correlation exists between parental phubbing and adolescents’ gratitude. Second, basic psychological needs’ satisfaction mediates the relationship between parental phubbing and gratitude. Finally, friendship quality moderates the first segment of the mediating pathway where parental phubbing impacts gratitude via basic psychological needs’ satisfaction, and it also moderates the negative indirect effect of parental phubbing on adolescents’ gratitude via basic psychological needs’ satisfaction. These findings emphasize the need to address the negative impact of parental phubbing on adolescents’ gratitude in intervention programs.

## Figures and Tables

**Table 1:** Descriptive statistics and correlation analysis of variables.

Variables	*M*	*SD*	1	2	3	4	5
1. T1 parental phubbing	3.04	0.74	1				
2. T1 basic psychological needs’ satisfaction	4.98	1.22	−0.45 **	1			
3. T1 friendship quality	3.84	0.66	−0.12 **	0.29 **	1		
4. T2 gratitude	4.56	0.89	−0.21 **	0.35 **	0.29 **	1	
5. T1 gratitude	4.57	0.90	−0.22 **	0.46 **	0.36 **	0.49 **	1

Note: ** *p* < 0.01.

**Table 2:** Mediation effect of basic psychological needs’ satisfaction.

	T1 Basic Psychological Needs’ Satisfaction				T2 Gratitude			
	*β*	*SE*	*t*	95% *CI*	*β*	*SE*	*t*	95% *CI*
T1 parental phubbing	−0.37	0.03	−11.07 ***	[−0.43, −0.30]	−0.05	0.04	−1.41	[−0.13, 0.02]
T1 basic psychological needs’ satisfaction					0.13	0.04	3.09 **	[0.05, 0.21]
*R* ^2^	0.34				0.26			
*F*	162.88 ***				74.45 ***			

Note: ** *p* < 0.01, *** *p* < 0.001.

**Table 3:** Moderated mediation effects of gratitude.

	T1 Basic Psychological Needs’ Satisfaction				T2 Gratitude			
	*β*	*SE*	*t*	95% *CI*	*β*	*SE*	*t*	95% *CI*
T1 parental phubbing	−0.35	0.03	−10.82 ***	[−0.42, −0.29]	−0.05	0.04	−1.41	[−0.13, 0.02]
T1 friendship quality	0.13	0.03	3.73 ***	[0.06, 0.19]				
T1 basic psychological needs’ satisfaction					0.13	0.04	3.09 **	[0.05, 0.21]
T1 parental phubbing × T1 friendship quality	−0.07	0.03	−2.60 **	[−0.13, −0.02]				
*R* ^2^	0.36				0.26			
*F*	88.94 ***				74.45 ***			

Note: ** *p* < 0.01, *** *p* < 0.001.

**Table 4:** Conditional direct effect.

	Effect	BootSE	95%BootLLCI	95%BootULCI
Low T1 friendship quality (M − 1SD)	−0.28	0.05	−0.37	−0.19
High T1 friendship quality (M + 1SD)	−0.43	0.04	−0.51	−0.35

**Table 5:** Conditional indirect effect.

	Effect	BootSE	95%BootLLCI	95%BootULCI
Low T1 friendship quality (M − 1SD)	−0.036	0.016	−0.071	−0.009
High T1 friendship quality (M + 1SD)	−0.055	0.022	−0.103	−0.015

## Data Availability

The data that support the findings of this study are available on request from the corresponding author.
